# Discovery of a New Analgesic Peptide, Leptucin, from the Iranian Scorpion, *Hemiscorpius lepturus*

**DOI:** 10.3390/molecules26092580

**Published:** 2021-04-28

**Authors:** Sedigheh Bagheri-Ziari, Delavar Shahbazzadeh, Soroush Sardari, Jean-Marc Sabatier, Kamran Pooshang Bagheri

**Affiliations:** 1 Venom and Biotherapeutics Molecules Laboratory, Medical Biotechnology Department, Biotechnology Research Center, Pasteur Institute of Iran, Tehran 1316943551, Iran; sebagheri2000@gmail.com (S.B.-Z.); shahbazzadeh@yahoo.com (D.S.); 2 Drug Design and Bioinformatics Unit, Medical Biotechnology Department, Biotechnology Research Center, Pasteur Institute of Iran, Tehran 1316943551, Iran; ssardari@hotmail.com; 3 Institute of NeuroPhysiopathology (INP), Faculté de Pharmacie, Université d’Aix-Marseille, UMR 7051, 27 Bd Jean Moulin, CEDEX, 13385 Marseille, France; sabatier.jm1@gmail.com

**Keywords:** Leptucin, analgesic peptide, *Hemiscorpius lepturus*, scorpion

## Abstract

*Hemiscorpius lepturus* scorpion stings do not induce considerable pain based on epidemiological surveys conducted in the southwest part of Iran. Accordingly, this study was aimed to identify the analgesic molecule in *H. lepturus* venom by analyzing a cDNA library of the scorpion venom gland looking for sequences having homology with known animal venom analgesic peptides. The analgesic molecule is a cysteine rich peptide of 55 amino acids. the synthetic peptide was deprotected and refolded. RP-HPLC, Ellman’s, and DLS assays confirmed the refolding accuracy. Circular dichroism (CD) showed helix and beta sheet contents. This peptide, called leptucin, demonstrated 95% analgesic activity at the dose of 0.48 mg/kg in hot plate assay. Leptucin at the doses of 0.32, 0.48, and 0.64 mg/kg showed 100% activity in thermal tail flick test. No hemolysis or cytotoxicity was observed at 8 and 16 μg. Histopathology evaluations indicated no hepatotoxicity, nephrotoxicity, and cardiotoxicity. We thus report that leptucin is the analgesic agent of *H. lepturus* venom. Regarding the high in vivo efficacy of leptucin and the fact it shows no observable toxicity, it could be suggested as a drug lead in a preclinical study of acute pain as well as the study of its mechanism of action.

## 1. Introduction

Pain is the most well-known unpleasant experience which protects a human from injuries [1,2]. Injuries cause acute or chronic pain. Acute pain occurs immediately after an injury and is usually severe in nature, whereas chronic pain continues beyond the normal healing process. It can start as acute pain but lasts for more than 3 months. The main causative agents for acute pain include surgery, traumas, bone fractures, dental work, burns or cuts, and for chronic pain lower back pain, cancer, and arthritis [3].

An analgesic is a drug that relieves pain [4]. They are classified into non-opioid analgesics (paracetamol, ibuprofen, COX-2 inhibitors, NSAIDs, diclofenac, etc.) and opioid analgesics (morphine, oxycodone, etc.) [5,6]. Among analgesic drugs, NSAIDs are routinely used for acute pain, but in the case of contraindication and severe pain, opioids are used [5]. Among opioids, morphine is the gold standard for pain relief, but their use is limited due to a number of side effects, including respiratory depression, constipation, intolerance, dependence, and addiction [7]. Thus, the design or discovery of a new molecule with a lesser complication is necessary. Since peptide molecules are more targeted and have fewer side effects than chemical small molecules, analgesic peptides could be a new revolution in pain-relief [8,9,10].

Venomous animals are important sources for discovery or development of new drug leads [11,12,13,14,15,16,17,18,19,20,21]. Approved peptide drugs from the venom of venomous animals include captopril (isolated from the venom of *Bothrops jararaca*, a pit viper, to control blood pressure), eptifibatide and tirofiban (isolated from viperid snake venom as an antiplatelet drug), lepirudin and bivalirudin (isolated from leech venom as anticoagulants), ziconotide (isolated from cone snails venom to suppress chronic pain), exenatide (isolated from lizard venom to treatment of type 2 diabetes) [15].

Venomous creatures have high potential for discovery or development of peptide drug leads [22,23,24]. Among the venomous animals, scorpions are also an important source for discovery of potential peptide or protein molecules with different pharmacological activities [24,25,26,27].

Some peptides in scorpion venom make scorpion stings very painful [28,29,30], but the stings of the Iranian scorpion, *Hemiscorpius lepturus* (*H. lepturus*) do not cause considerable pain based on local evidence, epidemiological studies, and clinical reports [31,32,33,34,35]. To our best of knowledge, no study has been conducted on the cause of the painless stings of this scorpion.

*H. lepturus* belongs to the hemiscorpidae family, distributed in Iran, Iraq, Pakistan, and Yemen [36]. To date, some peptides with pharmacological or toxicological activities have been discovered from the venom of *H. lepturus* venom i.e., hemicalcin [37], hemotoxin [36], hemilipin [38], PLD1 [31], HL2153 and HL2155 [39].

In the present study, we identified in the cDNA library of the *H. lepturus* scorpion venom gland a peptide homologue to a known analgesic peptide from sea anemone. The peptide was synthetized, refolded, and analyzed for its structure and analgesic activity as well as its toxicity.

## 2. Materials and Methods 

### 2.1. Materials, Cells, and Animals

3-(4,5-Dimethyl-2-thiazolyl)-2,5-diphenyl-2H-tetrazolium bromide (MTT) was purchased from Sigma (Saint Louis, MO, USA) and isopropanol, triflouroacetic acid (TFA), and acetonitrile (ACN) were purchased from Merck (Darmstadt, Germany). Morphine sulfate was obtained from Darou Pakhsh Pharmaceuticals (Tehran, Iran). DMEM and fetal bovine serum (FBS) were purchased from Gibco, Life Technologies (Grand Island, NY, USA). Human Embryo Kidney (HEK293, NCBI code C497) was obtained from Pasteur Institute of Iran (Tehran, Iran).

NMRI mice (Male, 25 g) were purchased from the Pasteur Institute of Iran. The animals were allowed to adapt for a week in standard conditions with a dark/light cycle of 12 h. The room temperature was 22 ± 1 °C and the relative humidity was adjusted at 50 ± 5%. The animals received a standard pellet diet and fresh tap water. All experiments were approved by the Ethical Committee of the Pasteur Institute of Iran (code number IR.PII.REC.1394.86).

### 2.2. Bioinformatics Analyses 

#### 2.2.1. Data Mining and ORF Determination

In this study, we used the data from a cDNA library of the venom gland of *H. lepturus* scorpion, which had been generated by Illumina RNA sequencing in 2017 [31], in order to find the analgesic molecule. The relevant keywords like analgesic, anti-pain, and pain relieving were searched in the annotated library. Then the corresponding DNA sequence was translated to ORF using ORF finder (https://www.ncbi.nlm.nih.gov/orffinder/) (accessed on 16 March 2020).

#### 2.2.2. Similarity Analysis and Determination of the Mature Chain

The similarity of ORF was determined against non-redundant and reference sequences using the ‘BLASTP’ server (http://blast.ncbi.nlm.nih.gov/Blast.cgi) (accessed on 17 March 2020). The mature chain and signal peptide of its similar sequences were checked in NCBI and UniProt. Then, for accurate determination of signal peptide and mature chain, the identified ORF for *H. lepturus* analgesic peptide was multiple aligned against the similar sequences using ‘COBALT’ server [40]. To control the accuracy of alignment, the evolutionary signature of aligned peptides was determined too. Then the molecular weight of the mature chain was calculated using the ‘Protparam’ server (http://web.expasy.org/protparam) (accessed on 18 March 2020).

#### 2.2.3. Prediction of 3D Structure of the Peptide

The 3D structure of the peptide was predicted using the Iterative Threading Assembly Refinement (I-TASSER) server at Michigan University [41]. A model with the lowest root mean square deviation (RMSD) was selected. This model was viewed and analyzed by using ‘UCSF Chimera’ software package [42]. Structural alignment with the analgesic peptide of a sea anemone, was performed and visualized using the UCSF Chimera software. Based on 3D structure analysis, disulfide bonds between cysteine amino acids were predicted.

### 2.3. Peptide Synthesis

The peptide was synthesized by an external facility (Biomatik Co., Cambridge, ON, Canada) using the solid phase method and Fmoc (9-fluorenyl-methoxycarbonyl) chemistry. In order to avoid oxidizing the sulfur moieties of cysteine, all cysteines in peptides were protected with acetamidomethyl (ACM) groups. The peptide was amidated at the C-terminal. The peptide was purified up to 95% by reverse phase-high performance liquid chromatography (RP-HPLC). The molecular weight of the peptide was also confirmed using the mass spectrometry method.

### 2.4. Deprotection of the Synthetic Peptide 

In order to refold the peptide and examine its activity, protected groups must be removed. Deprotection was performed based on the molar ratio method [43]. First, 1 mg of the synthesized peptide was dissolved in degassed distilled water. Then 100 μL of the peptide solution was added to 900 μL of mercury acetate solution (0.1% in degassed distilled water) and incubated at RT for 60 min. Then, 10 μL of 2-mercaptoethanol (10 mM) was added to the solution, incubated for 30 min under nitrogen gas, and centrifuged at 10,625× *g* for 5 min. The supernatant was collected and freeze-dried at −55 °C for 24 h in a freeze dryer system (alpha 1–2 LD plus; Martin Christ Gefriertrocknungsanlagen GmbH, Osterode am Harz, Germany).The abovementioned method was performed in four pH protocols. The peptide was dissolved in two pH conditions (i.e., 4 and 7) and the deprotection of peptides was subsequently performed in two pH conditions (i.e., 4 and 7).

#### 2.4.1. Quality Control of the Deprotected Peptide by SDS-PAGE

To compare the yield of peptide in the supernatant and pellet in four pH protocols, SDS-PAGE was performed using 15% polyacrylamide gel. The best protocol of deprotection method was considered for further evaluation in RP-HPLC.

#### 2.4.2. Evaluation of the Accuracy of the Deprotection Method by RP-HPLC 

RP-HPLC was carried out on a Knauer system (Knauer Wissenschaftliche Gerate Co., Berlin, Germany), with C18 column (Beckman-Coulter, Brea, CA, USA) 100 Å, 250 × 4.6 mm). One hundred μL of peptide solution was injected into the injector and a linear gradient of acetonitrile was applied from 0 to 90% (absolute acetonitrile containing 0.1% TFA) at a flow rate of 1 mL/min for 90 min. After that, the retention time of the deprotected peptide was compared to the protected one. Any alteration in the retention time of the deprotected peptide indicates that the peptide is deprotected. The deprotected peptide was collected and lyophilized as mentioned above.

#### 2.4.3. Determination of the Purity and Yield of the Deprotected Peptide

The purity of the deprotected peptide was determined by using chromgate software package. The yield was determined by calculation of the difference between the amount of peptide before and after deprotection. The concentration of the peptide was measured by UV estimation at 280 nm in a spectrophotometer. Yield percent was calculated using the following formula [30]:

(1) $$\mathrm{Yield}\;\mathrm{percent}\;=\;\left(\frac{\mathrm{peptide}\;\mathrm{amount}\;\mathrm{after}\;\mathrm{deprotection}\;}{\mathrm{peptide}\;\mathrm{amount}\;\mathrm{before}\;\mathrm{deprotection}}\right)\times 100$$

### 2.5. Refolding of the Deprotected Peptide

At first, ammonium acetate solution (20 mM, pH 6.5) prepared in 900 μL degassed water for injection (WFI). Then, the different amounts of reduced glutathione (GSH) and oxidized glutathione (GSSG) were added to each vial to reach a ratio of GSH/GSSG as 10:1 (3.07 mg: 0.612 mg) and 2:1 (0.614 mg: 0.612 mg). Ultimately, two amounts of deprotected peptide (40 and 80 μg in 50 and 100 μL, respectively) were added to the separate solutions. The final volume was adjusted to 1 mL by ammonium acetate solution (20 mM, pH 6.5).Then the solutions were stirred at 22 °C for 4 h [44]. All steps were performed under nitrogen gas to ovoid oxidizing the cysteine amino acid residues.

To determine the optimal refolding conditions, RP-HPLC was performed for the sample and negative control (peptide-free buffer) as well. The results were compared to negative control, and the peak corresponding to the refolded peptide was collected and lyophilized. SDS-PAGE was then performed to verify the existence of peptide. Then, the resulting peptide was subjected to Ellman assay to further confirmation of peptide refolding. For the activity assays, the sample was dialyzed against normal saline in a dialysis tube (MWCO: 3000 Da) for 24 h.

#### 2.5.1. Determination of the Purity and Yield of the Refolded Peptide

The purity of the refolded peptide was determined by using chromgate software package. The yield was determined by calculation of the difference between the amount of peptide before and after refolding. The concentration of the peptide was measured by UV estimation at 280 nm in a spectrophotometer. Yield percent was calculated as detailed above [30].

#### 2.5.2. Confirmation of Refolding by Ellman’s Assay

This assay was performed to confirm that there is no free thiol residue in the surface of refolded peptide. No reaction is expected for the refolded peptide. This assay was performed based on the protocol developed by Shahangian, et al. [45].

Briefly, refolded peptide (5 mM) incubated with a reaction mixture (8M urea, 100 mM sodium-phosphate, pH 8.0, and 1 mM 5,5′-dithiobis-(2-nitrobenzoic acid) (DTNB)) at room temperature. The optical density (OD) was monitored at 412 nm after 1 h. The Ellman’s reagent solution without refolded peptide was used as negative control. The number of free thiols was calculated using a molar extinction coefficient of 13,600 M^−1^ cm^−1^.

### 2.6. Dynamic Light-Scattering (DLS) 

To assess the homogeneity of the refolded purified peptide (100 μg in 500 μL water/TFA 0.05%), DLS analysis was performed at RT and a scattering angle of 90° using a Zetasizer Nano-ZS instrument (ZEN 3600, Malvern Co., Malvern, UK). A typical protein refractive index of 1.45 was used.

### 2.7. Circular Dichroism

The secondary structure of the refolded peptide was studied by circular dichroism (CD) spectroscopy using a spectropolarimeter (JASCO J-810, JASCO International Co., Ltd., Tokyo, Japan). At first, the refolded peptide (200 μg) was resuspended in 300 μL water/TFA 0.05%. The sample was placed into a 0.1 cm path-length quartz Jasco cell at 25 °C. The spectra were recorded in the range of 190–260 nm with averaging three scans. The CD spectra of peptide free buffer are also recorded as control similarly. The percent of alpha helix, beta sheet, turn, and coil conformations was calculated using the Spectra Manager^TM^ II software package.

### 2.8. The Evaluation of Analgesic Activity for the Refolded Leptucin

#### 2.8.1. Hot Plate Test

This assay was performed to investigate the central analgesic effect [46]. Briefly, the mice were first placed on 55 °C hot plate and pain responses (hind paw withdrawal, licking and/or shaking) were recorded. Mice with normal pain responses were selected and randomly divided into six groups (four different doses of peptide, negative control, and positive control); six mice in each group.

The peptide at the doses of 0.16, 0.32, 0.48, and 0.64 mg/kg (4, 8, 12 and 16 μg/mouse) were administrated intraperitoneally. Normal saline and morphine (10 mg/kg) were used as negative and positive control, respectively.

Reference to FDA guidance for testing analgesics, all analgesics has characteristics that create a challenge for clinical trial design. Pain is a subjective phenomenon and often fluctuates over time. Pain intensity changes over a relatively short period of time, which can present challenges in designing a trial. Pain intensity can be measured by numerical rating scales, visual analog scales, or categorical scales [47] (http://www.fda.gov/Drugs/GuidanceComplianceRegulatoryInformation/Guidances/default.htm) (accessed on 18 February 2021).

Thus, we followed FDA guidance as a realistic approach to recording pain responses. Accordingly, the peptide efficacy was determined as the percent of activity instead of recording latency.

After 30 min, the pain responses in each group were recorded. A cut-off time was set at 40 s. The time periods to recording pain responses were determined at thirty-minute intervals up to 300 min after administration. Analgesic activity was calculated at each time point [48].

#### 2.8.2. Tail Flick Test

This test was performed using a tail flick apparatus (Pooya Armaghan Co., Tehran, Iran) to evaluate central analgesic effect [49,50]. The mice were placed horizontally in the mice enclosure, their tails (5 cm from the tip) exposed to the heat radiant, and the tail retraction time was recorded. The baseline latencies of the radiant heat were set to 2.5–3.5 s to avoid tissue damage and the cut-off latency was adjusted to 10 s. The mice were randomly divided into 6 groups; four doses of peptide, negative control, and positive control. Normal saline and morphine (10 mg/kg) were used as negative and positive control, respectively. The peptide at the doses of 0.16, 0.32, 0.48, and 0.64 mg/kg (4, 8, 12 and 16 μg/mouse) were administrated intraperitoneally. Then, the latency of the tail reaction in each group was measured before the injection of the peptide or negative and positive controls. Thirty minutes after injection, the tail retraction time was recorded.

### 2.9. Toxicity Tests for Leptucin

#### 2.9.1. MTT Test

In order to determine the cytotoxic effect of peptide, MTT assay was done on HEK-293 cell line. The cells (2 × 10^4^) were plated in 96-well plates in DMEM medium, supplemented with 10% FBS and then incubated in the atmosphere containing 5% CO_2_ and 95% air at 37 °C for overnight. The peptide, at the concentrations ranging from 16 to 0.25 μg/mL, was prepared in DMEM medium, added to each well, and incubated for 24 h. A 10 μL solution of freshly prepared MTT (5 mg/mL in PBS) was added to each well and incubated for an additional 4 h. Isopropanol (100 μL) was then added and the plates were shaken gently to facilitate formazan solubilization. The absorbance was measured at 570 nm by an ELISA reader (ELx808, BioTek, Winooski, VT, USA), and the viability was then evaluated [51].

#### 2.9.2. In Vitro Hemolysis Assay

This assay was performed according to Memar et al.’s protocol [52]. Briefly, human blood from a healthy volunteer was collected, centrifuged at 664× *g* for 5 min, and washed with PBS three times.

Two doses of peptide (8 μg and 16 μg) were prepared in 100 μL of PBS (1×). One hundred μL of washed RBCs suspension (2%) was added to each well of a 96-well microplate (Nunc, Sigma Co., St. Louis, MO, USA). Phosphate buffer saline and Triton X-100 (0.1%) was used as negative and positive control, respectively. The microplate was incubated at 37 °C for 2 h and centrifuged at 1664× *g* for 10 min. The supernatant was transferred to a new plate and OD was measured at 540 nm in a microplate spectrophotometer (EPOCH, BioTek, Winooski, VT, USA). The experiment was carried out in triplicate. The degree of hemolysis was determined as the following formula:

[(OD sample − OD neg control)/(OD pos control − OD neg control)] × 100(2)

#### 2.9.3. In Vivo Hemolysis Assay

Three doses of peptide, 0.32, 0.48, and 0.64 mg/kg (8, 12, and 16 μg/mouse), were injected intraperitoneally and the blood samples were collected after 24 and 48 h. The blood samples were centrifuged at 1180× *g* for 5 min, and the absorbance of supernatant was measured at 540 nm by a microplate spectrophotometer (EPOCH, BioTek, Winooski, VT, USA) [18]. Normal saline was used as negative control.

#### 2.9.4. Histopathological Study

Two doses of peptide, 0.32 and 0.64 mg/kg (8 and 16 μg/mouse), were injected intraperitoneally, and the mice were sacrificed after 48 h of peptide administration. Liver, kidney, and heart were collected and fixed in 10% buffered formalin solution. After 4 days, the organ sections were routinely processed in an automated tissue processor and embedded in paraffin wax. The tissue sections were prepared and stained with hematoxylin and eosin (H&E) for pathological studies [53].

#### 2.9.5. Determination of Lethal Dose 50

Lethality due to leptucin was examined on BALB/c mice (male, 20–30 g) using routine method. Leptucin at the doses of 0.8, 1.6, 2.4, 3.2, and 4 mg/kg were prepared in 100 μL sterile PBS (1×) and injected intraperitoneally into each group (6 mice/group). Sterile PBS (1×) was used as negative control.

### 2.10. Motor Coordination Test (Rotarod Test)

The animals are placed on textured drums (1¼ inch diameter) to avoid slipping. When an animal drops onto the individual sensing platforms, the result is recorded. Five mice were tested at a rate of 4 rpm.

Naive mice were trained until they could remain on the rotarod for 5 min. Animals that failed to meet this criterion within three trials were discarded.

Mice were intraperitoneally pretreated with either normal saline or leptucin at the doses of 0.32, 0.48, and 0.64 mg/kg (8, 12, and 16 μg/mouse). Thirty minutes after the injection of peptide, mice were placed on the rotarod for 5 min. If a mouse fell from the rotarod during this time period, it was scored as motor impaired. The data was subjected to ANOVA followed by Student’s t-test [54].

### 2.11. Data Analysis

Data obtained from the two pain models tests are expressed as mean ± SD. Normality was assessed using the Kolmogorov-Smirnov test. The significance of the effect was tested by one-way analysis of variance (ANOVA) followed by Tukey’s post hoc analysis. In all cases, the results were considered significant where *p* < 0.05. Effect dose of 50% (ED50) was measured by linear regression assay. LD50 was determined within 72 h following *i.p.* administration of leptucin; six mice in each group.

## 3. Results 

### 3.1. Identification of Mature Chain and Sequence Analysis

**Figure 1:**
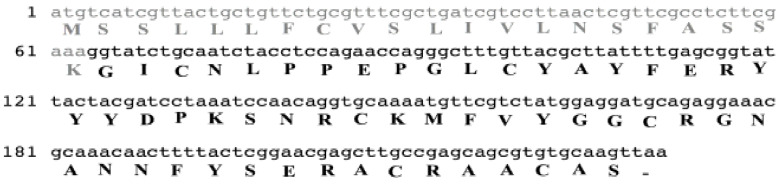
The amino acid and ORF sequence of leptucin; the *H. lepturus* analgesic peptide. The area of the signal peptide and mature chain are colored gray and black respectively.

**Figure 2:**
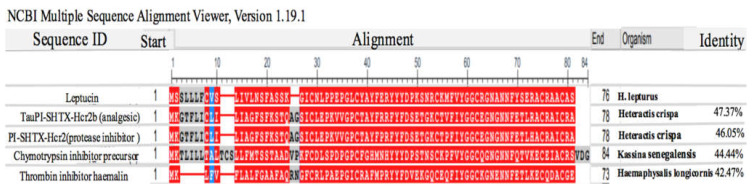
Multiple alignment of leptucin with selected similar peptides by COBALT server. Leptucin’s ORF was compared to its closest similar peptides. As shown in the figure, that was similar to different peptides with identity ranging from 47.37 to 42.47%. Also, analysis showed a conserved signature sequence in the selected similar sequences as [C (6XGX) C (8XYXD5X) C (2XFXYGG) C (XGNXNNF5X) C (3X) C (2X)].

According to the data mining analyses, an 859 bp sequence corresponding to the annotation of analgesic peptide was concluded in the cDNA library. Analysis of the raw sequence in ORF finder server showed a 231 bp sequence encrypted as a peptide of 76 amino acids (Figure 1). Analysis of sequence similarity by blastP showed that the *H. lepturus* analgesic peptide was similar to different peptides with identity ranging from 47.37 to 42.47% (Figure 2). TauPI-stichotoxin-Hcr2b (UniProt P0DMJ5, AltName: APHC1), an analgesic peptide from the sea anemone; *Heteractis crispa*, had the highest similarity (Identity 47%) to *H. lepturus* analgesic peptide (Figure 2). The obtained result reconfirmed the accuracy of annotation analysis of cDNA library of *H. lepturus* venom gland.

Multiple alignment analyses showed a conserved signature in the selected similar peptides as [C (6XGX) C (8XYXD5X) C (2XFXYGG) C (XGNXNNF5X) C (3X) C (2X)]. In all selected similar peptides, the signal peptide consisted of 22 amino acids, except for one, which were 15 amino acids.

The sequence comparison between *H. lepturus* analgesic peptide and similar peptides led to determination of the mature chain in *H. Lepturus* analgesic peptide. The mature chain is a cysteine-rich peptide which contains 55 amino acids (Figure 1), hereafter designated as leptucin and its theoretical molecular weight was calculated as 6262.09 Da.

### 3.2. Prediction of 3D Structure of the Peptide

**Figure 3:**
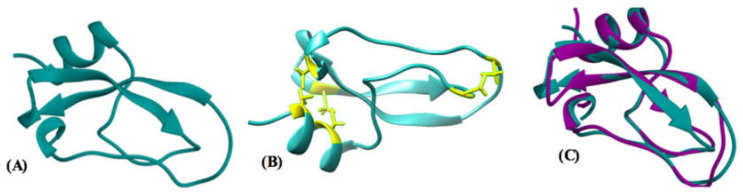
(**A**) Prediction of the 3D structure of leptucin. Analysis of the result showed that the peptide comprises alpha helices (25.45%), beta sheets (25.45%), and random coil (49.09%). (**B**) Based on 3D structure, disulfide bonds between cysteine amino acids were predicted in leptucin as C_1_-C_6_*C_2_-C_4_*C_3_-C_5_. (**C**) Superimposition of the structure of TauPI-stichotoxin-Hcr2b with the predicted structure of leptucin.

The predicted structure of leptucin contains two β sheets and one α helix (Figure 3A). Disulfide bonds between cysteine amino acids were determined by UCSF Chimera software packages (Figure 3B). Structural alignment of leptucin with TauPI-stichotoxin-Hcr2b [55] by UCSF Chimera software package showed a significant homology (RMSD = 1.085) as depicted in Figure 3C.

### 3.3. Deprotection Analysis 

Based on the molar ratio deprotection method, the peptide dissolved in pH 4 and after that added to deprotection solution (pH 4, protocol 1). SDS-PAGE was performed on the supernatant and pellet to control the efficiency of the protocols. The results showed that the peptide was degraded and no band was seen. It was speculated that deprotected peptide would be degraded in this pH. In order to overcome this issue, different protocols were used as detailed in Table 1.

**Figure 4:**
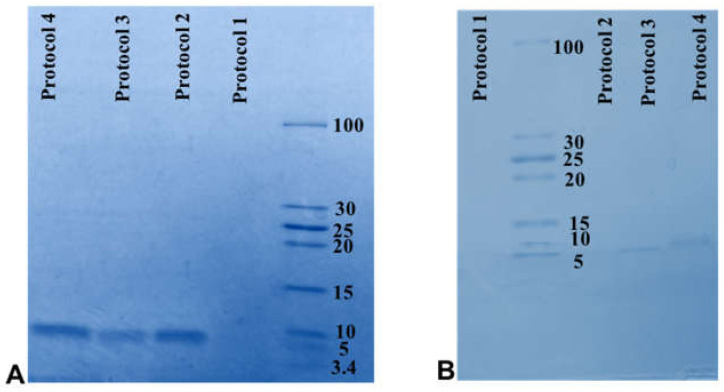
SDS-PAGE performed on the pellets and supernatants. (**A**) SDS-PAGE on the supernatant showed that the peptide destroyed in protocol 1. The result showed that the peptide amount obtained in protocol 2 (peptide in pH 4, deprotection in pH 7) and 4 (peptide in pH 7, deprotection in pH 4) were more than protocol 3 (peptide in pH 7, deprotection in pH 7). (**B**) SDS-PAGE on the pellets showed that peptide band did not exist in protocol 1 (peptide in pH 4, deprotection in pH 4), but in protocol 3 (peptide in pH 7, deprotection in pH 7) and protocol 4 (peptide in pH 7, deprotection in pH 4) there were a little deprotected peptide.

SDS-PAGE was finally performed on the supernatants and pellets of all 3 protocols. The SDS-PAGE results showed that the band density in the supernatant of protocol 2 and 4 is approximately the same and was greater than protocol 3 (Figure 4A,B). There was a thin deprotected peptide band in the pellet of protocol 4, but no band was seen in the pellet of protocol 2. According to the results, protocol 2 was considered the optimal protocol.

### 3.4. The Accuracy of Deprotection 

**Figure 5:**
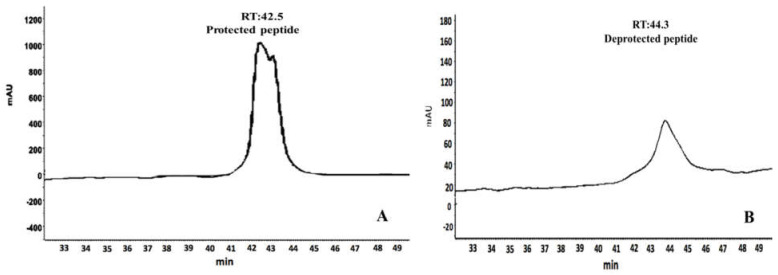
Comparison of the retention time between deprotected and protected in RP-HPLC. ACM protected (**A**) and deprotected (**B**). The retention time after deprotection shifted 1.8 min to the right.

To confirm the accuracy of deprotection, RP-HPLC was performed. One hundred μL of the peptide (in supernatant), collected from the best deprotection protocol, was injected onto a C18 column. RP-HPLC analysis showed that the retention time of the deprotected peptide (Figure 5A) had shifted 1.8 min to the right (Figure 5B) in comparison to the protected peptide.

One major peak was seen in the chromatogram and its purity calculated as 92% by chromgate software (Figure 5B). A total of 114 μg deprotected peptide recovered from 300 μg protected one, indicating the yield as 38%.

### 3.5. Refolding Analysis 

**Figure 6:**
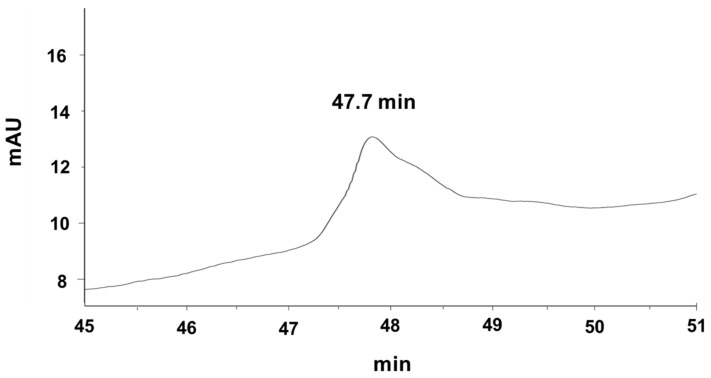
RP-HPLC for the refolding peptide. The refolding of 80 μg peptide in the ratio GSH2/GSSG1 concluded the best result in which just one peak was observed.

After refolding of the deprotected peptide in the different ratio of GSH/GSSG, RP- HPLC was performed. The results show that there isn’t any peak of refolded peptide at the amount of 40 μg in the ratios of GSH2/GSSG1 and GSH10/GSSG1 in comparison to negative control. After changing the amount of peptide to 80 μg, in a ratio of GSH2/GSSG1 a sharp peak was observed. The amount of 80 μg peptide in a ratio of GSH10/GSSG1 concluded two peaks (Figure S1). The refolding of 80 μg peptide in the ratio of GSH2/GSSG1 concluded the best result in which just one peak was observed (Figure 6).

Analysis of the RP-HPLC results showed that the retention time of the refolded peptide had shifted 3.4 min to the right in comparison to the deprotected peptide.

The purity of the refolded peptide was calculated as 90% by chromgate software. A total of 108.3 μg refolded peptide recovered from 114 μg deprotected one. Based on the calculation, the yield of refolding was reported as 95%.

### 3.6. Refolding Confirmation

**Figure 7:**
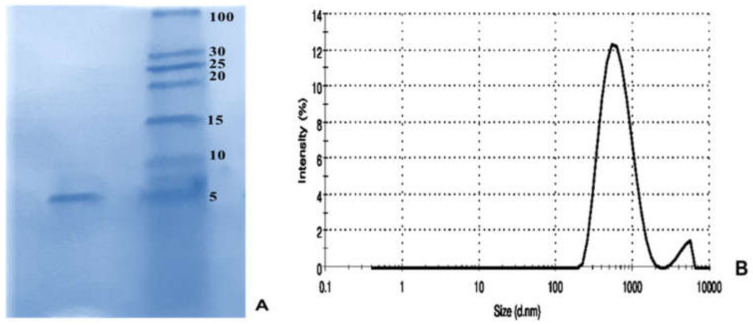
Refolding confirmation. (**A**) SDS-PAGE for the refolded leptucin, (**B**) Homogeneity evaluation of the refolded peptide using dynamic light scattering.

The refolded peptide was shown by SDS-PAGE (Figure 7A). According to the result, obtained from Ellman’s assay, no free thiol residue was detected in the refolded peptide indicating all cysteine residues participated in the formation of disulfide bridges in the peptide. The homogeneity of refolded peptide was analyzed using DLS. The result showed that the homogeneity in the refolded peptide was 95.6% (Figure 7B).

### 3.7. Circular Dichroism

**Figure 8:**
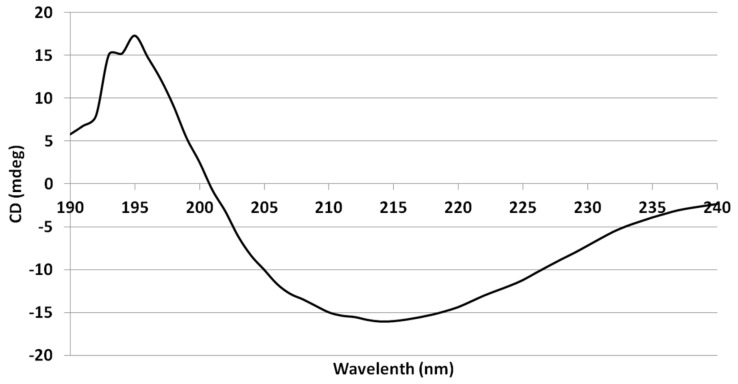
The CD spectra of leptucin. Analysis of the results showed that the peptide comprises alpha helices (20.4 %), beta sheets (43.1%), turn (11.6 %), and random coil (25%).

The CD spectrum of leptucin showed a negative band in the 208–222 nm region, which is characteristic of an α-helical structure. Also, one broad negative band around 217 nm and a shoulder around 195 nm were observed corresponding to a β-sheet structure (Figure 8). The proportions of α helices, β strands, turns, and random coils were calculated to be equal to 20.4, 43.1, 11.6, and 25%, respectively.

### 3.8. Activity Assays

#### 3.8.1. Hot Plate Assay

**Figure 9:**
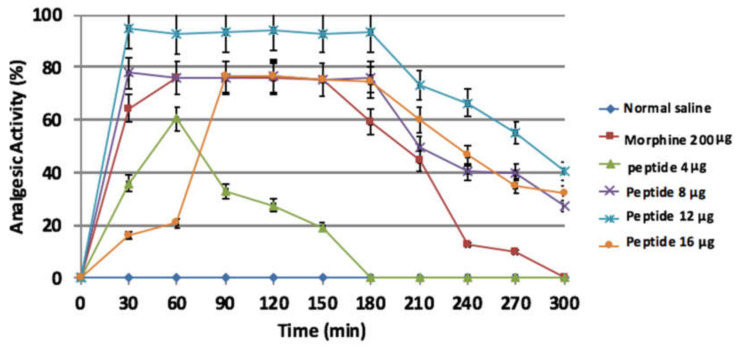
Hot plate assays. The maximum analgesic activity was 95% at 0.48 mg/kg (12 μg/mouse) after 30 min and continued up to the time point of 180 min. Data is expressed as mean ± SD. All groups had n = 6.

The mice were treated with peptide at the amount of 0.16, 0.32, 0.48, and 0.64 mg/kg (4, 8, 12, and 16 μg/mouse) or morphine at 10 mg/kg (200 μg/mouse). The results showed a significant increase in the analgesic effect in comparison to negative control (*p* < 0.0001), with the exception of 0.16 mg/kg. The maximum analgesic effect (activity 95%) for the peptide was obtained at the dose of 0.48 mg/kg (12 μg/mouse) after 30 min and continued up to the time point of 180 min. No differences were detected between peptide-treated groups of 0.32 and 0.64 mg/kg (*p* > 0.05) (Figure 9). The ED_50_ value was equal to 0.19 mg/kg (4.9 μg/mouse).

#### 3.8.2. Tail Flick Assay

**Figure 10:**
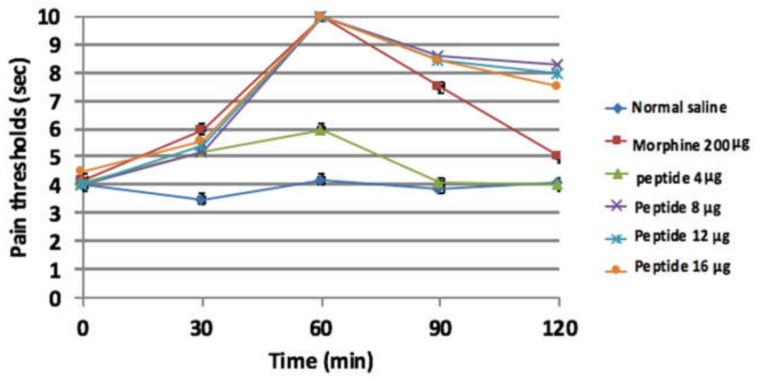
Tail-flick assay. The mice were treated by leptucin or morphine. The maximum analgesic activity of leptucin was obtained after 60 min at the doses of 0.32, 0.48, and 0.64 mg/kg (8, 12, and 16 μg/mouse). This result was similar to morphine (*p* > 0.05). Data are expressed as mean ± SD. All groups had n = 6.

Based on statistical analyses by using ANOVA, the peptide significantly increased the pain threshold at the doses of 0.16, 0.32, 0.48, and 0.64 mg/kg (4, 8, 12, and 16 μg/mouse), and there were significant differences between the test and control groups (*p* < 0.0001). The maximum analgesic effect of peptide was obtained at the doses of 0.32, 0.48, and 0.64 mg/kg after 60 min (*p* > 0.05). The latency of pain threshold continued at the doses of 0.32, 0.48, and 0.64 mg/kg for 120 min but did not last for morphine (Figure 10). The ED_50_ value of leptucin was equal to 0.17 mg/kg (4.3 μg/mouse).

### 3.9. Toxicity Assays

The analgesic peptide showed no toxicity on HEK293 cells at all examined concentrations (Figure S2). No toxicity was seen on human RBCs at the doses of 8 and 16 μg (Table S1). The results obtained from the in vivo hemolysis assay indicated no toxicity after 24 and 48 h at all examined doses (Table S2). Histopathology evaluation of liver, kidney, and heart showed that administration of analgesic peptide induces no hepatotoxicity (Figure S3), nephrotoxicity (Figure S4) and cardiotoxicity (Figure S5) up to 0.64 mg/kg (16 μg/mouse). LD_50_ value of leptucin was >4 mg/kg (100 μg/mouse) (Table S3).

### 3.10. Influence of Leptucin on Motor Coordination

In comparison to control group, leptucin had no effect on motor coordination (*p* > 0.05) which means that the peptide did not cause motor deficits in mice.

## 4. Discussion 

NSAIDs and morphine are the most common analgesic in acute pain [5], but their use is limited due to many side effects [7]. Concerning this issue, the design or discovery of a new molecule with lesser complication is necessary [8,9].

Using a screening strategy and bioinformatics analysis, we identified a sequence of an analgesic peptide in the annotated cDNA library of *H. lepturus.* Using multiple alignment analyses, the mature chain of peptide was determined and designated as Leptucin, which is a cysteine-rich peptide. Moreover, for the first time, an evolutionary signature was also determined [i.e., C (6XGX) C (8XYXD5X) C (2XFXYGG) C (XGNXNNF5X) C (3X) C (2X)] in the peptide sequence referring to its considerable similarity with other peptides in the Kunits-type cysteine-rich peptide family.

3D prediction showed that the peptide comprises α helices (25.45%), β sheets (25.45%), and random coil (49.09%) where the amounts of α helices, β strands, turns, and random coil were calculated to be equal to 20.4, 43.1, 11.6, and 25% respectively. The results obtained from 3D prediction and CD analysis thus did not match.

Preliminary analysis based on structural prediction and observation of structural similarity indicated that refolding is necessary for analgesic activity of leptucin.

In all deprotection methods, from the earliest established by Veber on cysteine-rich peptides [56], and then the modified Veber et al.’s protocol (Becker et al.’s protocol) [57], to the method of Eisapoor et al., which was the best method of deprotection [43], it’s a common point. In these methods, the removal of the ACM group with mercury acetate had been performed in pH 4 [43,56,57].

In this study, we used the molar ratio protocol for deprotection of leptucin because of its high yield, rapid and cost-effective advantages [43]. Our result showed that the peptide is destroyed in pH 4 after one day. Thus, to overcome this problem, we examined four pH protocols as mentioned in Table 1. Although deprotection could be performed immediately without peptide degradation, but in terms of stability it is better to reach an optimized protocol to avoid degradation during solubilization and storage.

According to the result, the peptide was seen in the collected supernatant in protocol 2. This finding is not in agreement with Becker et al., Veber et al., and Eisapoor et al.’s protocols as they had found the peptide in the precipitate. This finding can be so interesting in terms of industrial parameters. Being soluble in supernatant is cost-effective since the steps and the time for deprotection are decreased.

Among refolding methods, the methods based on the implementation of reduced and oxidized glutathione and guanidine chloride induce correct refolding in comparison to the other methods [44,58]. According to a 2006 study by Edward et al., guanidine is denaturing and can disrupt the three-dimensional structure of peptides. Thus, we preferred to use reduced and oxidized glutathione for refolding [59]. Refolding plays an important role in the stability and activity of the cysteine-rich peptides [60].

The refolding of cysteine-rich peptides depends on the peptide concentration and the ratio of GSH and GSSG. Different ratios of GSH and GSSG are used to refold different peptides [60,61]. In this study, a ratio of GSH10:GSSG1 and a minimum ratio of 2:1 with 40 and 80 μg of peptide were considered. The results showed that the ratio of 2:1 with 80 μg of peptide is the optimal molar ratio. The refolding was quite economical in terms of consuming reagents. The refolded peptide in this ratio showed one peak and shifted to the right in comparison to the deprotected peptide as shown in RP-HPLC. Using a ratio of GSH2: GSSG1, our study is in agreement with Lopez-Vera et al., and Buczek, et al. [42,58]. Based on Ellman’s result, it can be concluded that there is no free thiol moiety in the surface structure of leptucin and the refolding is done correctly. Based on the results obtained from DLS, the refolded peptide is mono-dispersed and homogenous [62].

Analgesic effects of leptucin were assessed using two well-known models of pain including hot plate and tail flick assays. Leptucin, at the doses of 0.32 and 0.48 mg/kg (8 and 12 μg/mouse) demonstrated maximum analgesic effect in tail-flick and hot plate assays, respectively. Analysis of the data obtained from tail flick and hot plate assays showed that leptucin has about 31.25- and 20.83-fold greater activity than morphine. Thus, it can be deduced that leptucin has more specificity towards the responsible channels for pain. This issue demonstrates that the peptide can exert less or no toxicity in comparison to morphine. The primary pathological evaluations and survival assay showed no toxicity in terms of acute toxicity. This fact may raise the hope for using this peptide for relieving acute pain as well as chronic pains, however, comprehensive toxicity studies should be performed.

According to the hot plate assay, the maximum effect of leptucin (activity 95%) was observed 30 min after administration and its maximum activity was constant for 150 min whereas in morphine group, the maximum effect (76.2%) was constant for 90.

As shown in the tail-flick assay, the maximum activity (100%) for leptucin was obtained after 60 min. The activity decreased slowly to 86% and was constant up to 120 min. In the morphine group, the maximum activity of 100% was seen after 60 min, but the activity decreased slowly to 0% up to 120 min.

Morphine is generally accepted to act at supraspinal sites or directly on spinal opioid receptors [63]. Thus, analgesic activity of leptucin in both of hot plate and tail-flick tests suggests that leptucin may act at supraspinal sites and directly on spinal opioid receptors too. Tail flick and hot plate assays are the most common tests of nociception. The nociceptive experience is short-lasting in acute pain models [2]. Therefore, it is suggested that Leptucin has an analgesic effect in an acute pain model.

Based on the analgesic activity and the sequence similarity, it would be interesting to test leptucin activity on ion channels of nociception TRPV1 [55].

Our result showed that leptucin at the dose of 8 and 16 μg had no toxicity on human red blood cells. In vivo hemolysis assay on mice demonstrated no hemolysis too.

Also, the peptide had no toxicity on HEK293 cell line by MTT assay up to 16 μg. Moreover, histopathological sections of liver, kidney, and heart demonstrated that Leptucin had no toxicity up to 0.64 mg/kg.

## 5. Conclusions

We discovered that leptucin is the analgesic agent of *H. lepturus* venom. Optimization of pH in deprotection steps led to obtaining the deprotected peptide in the supernatant. This point is important in terms of cost-effectiveness in the laboratory and industrial scale. According to the results gathered in Ellman’s test, DLS, and spectropolarimetry, the peptide was refolded successfully.

Regarding the high in vivo efficacy of leptucin and showing no observed toxicity, it could be suggested as a drug lead. Further studies should be performed to decipher more details about its action mechanism. Since peptide molecules can target specific channel and have fewer side effects than chemical small molecules, leptucin would be a new hope in pain-relief.

## Figures and Tables

**Table 1:** Different pH in the molar ratio deprotection method.

	Dissolution	pH	
Deprotection		4	7
pH	4	Protocol 1	Protocol 3
	7	Protocol 2	Protocol 4

## Data Availability

The data presented in this study are available in the manuscript.

## Supplementary Materials

The following are available online, Figure S1: RP-HPLC for the refolding peptide, Figure S2: MTT assay for leptucin, Figure S3: The histopathology evaluation of liver, Figure S4: The histopathology evaluation of kidney, Figure S5: The histopathology evaluation of heart. Table S1: In vitro hemolysis assay for leptucin, Table S2: In vivo hemolysis for leptucin, Table S3: Determination of LD50 for leptucin.

## Sample Availability Statement

Samples of the compounds are not available from the authors.
