# Supplementary material for: Discovery of a New Analgesic Peptide, Leptucin, from the Iranian Scorpion, Hemiscorpius lepturus
Source: Molecules. 2021 Apr 28;26(9):2580. doi: 10.3390/molecules26092580 (PMC8124257; doi:10.3390/molecules26092580)
Supplement: Supplementary file 1 [file molecules-26-02580-s001.zip › Supplementary.pdf]

Supplementary Figures and Tables

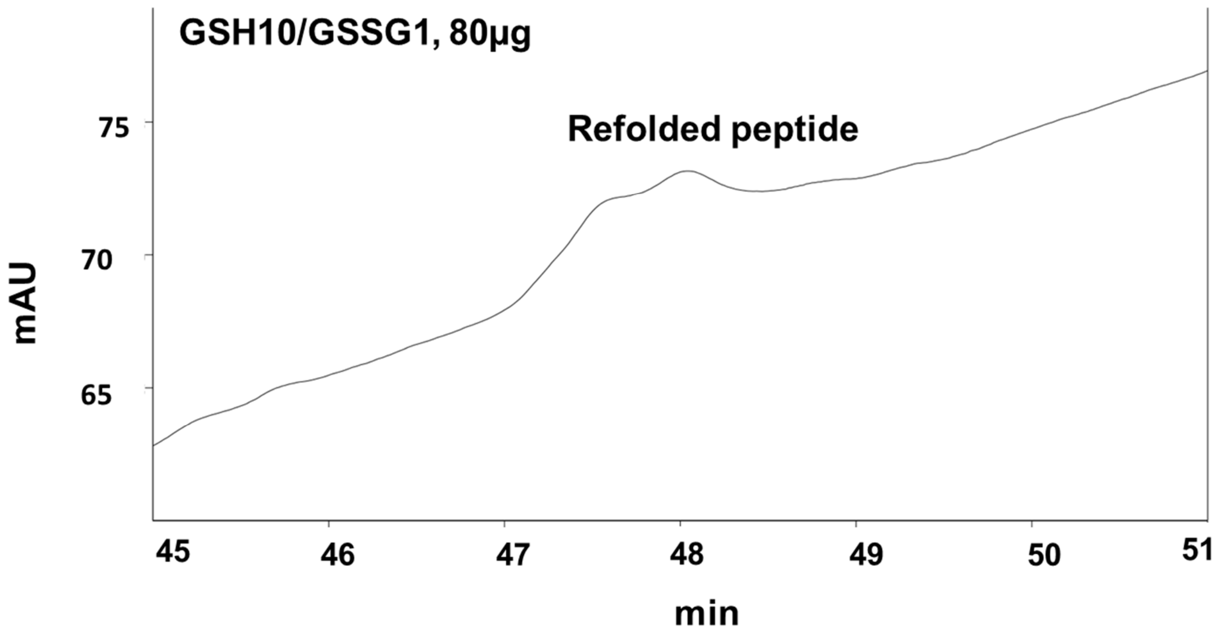

Figure S1. RP-HPLC for the refolding peptide. The amount of 80 μg peptide in a ratio of GSH10/GSSG1 concluded two peaks. This ratio negatively effects on the purity and yield of the refolded peptide.

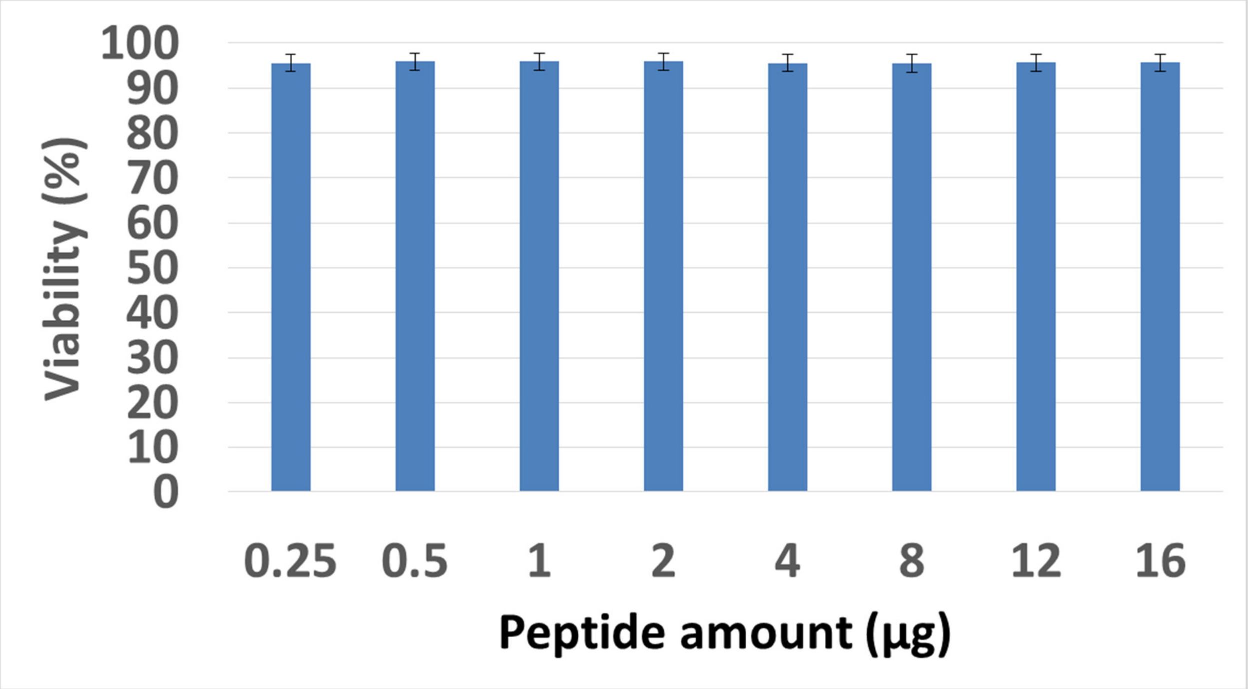

Figure S2. MTT assay for Leptucin. The analgesic peptide showed no toxicity on HEK293 cells at all examined concentrations.

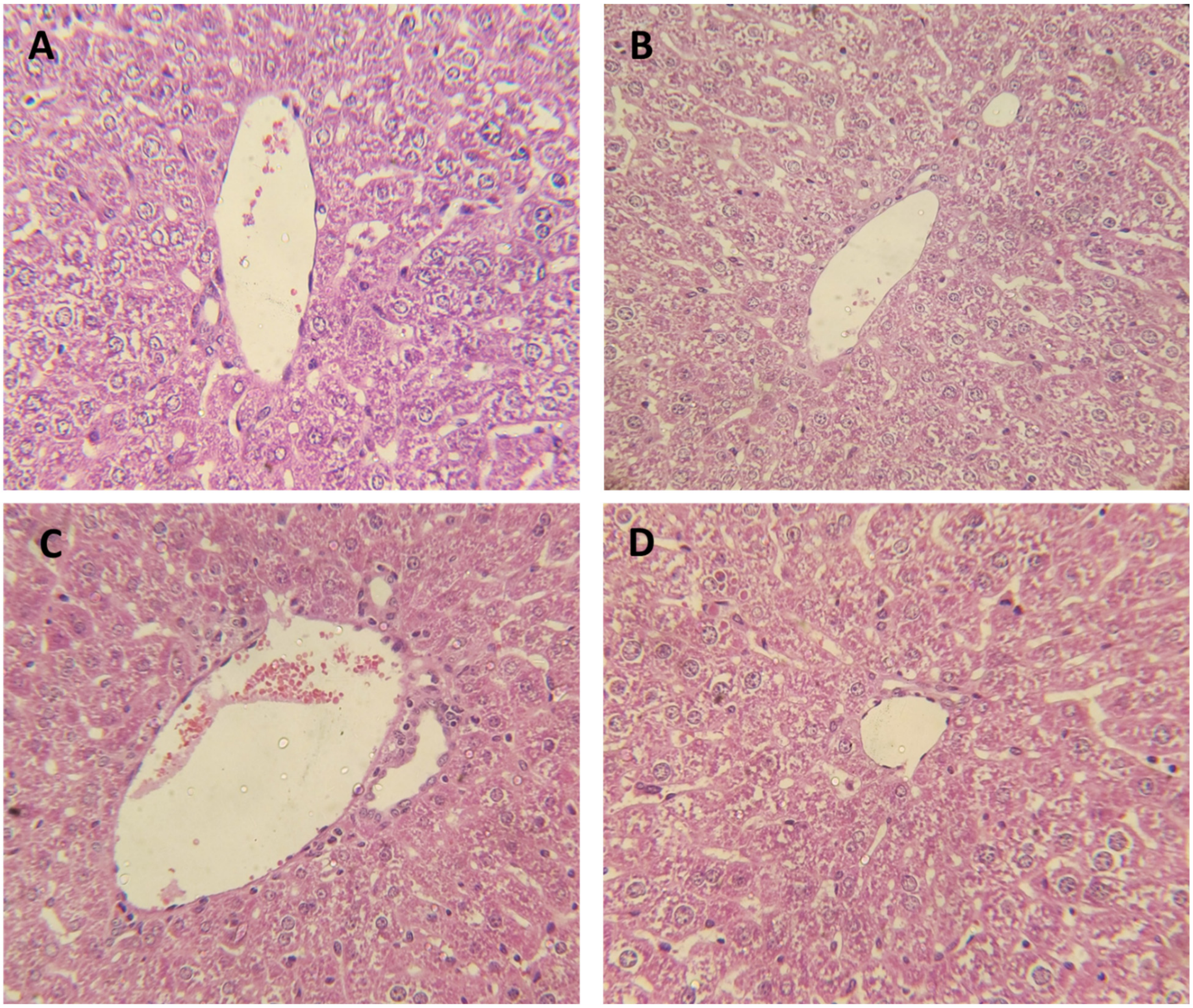

Figure S3. The histopathology evaluation of liver. Pathological events including central vein congestion, sinusoidal congestion, hydropic degeneration, hemorrhage, infiltration of inflammatory cells, increasing the numbers of kupffer cells, necrosis, and apoptosis were evaluated. Analysis of the results showed that administration of analgesic peptide induces no hepatotoxicity at the doses of 0.32 (**A**) and (**B**) and 0.64 mg/kg (**C**) and (**D**).

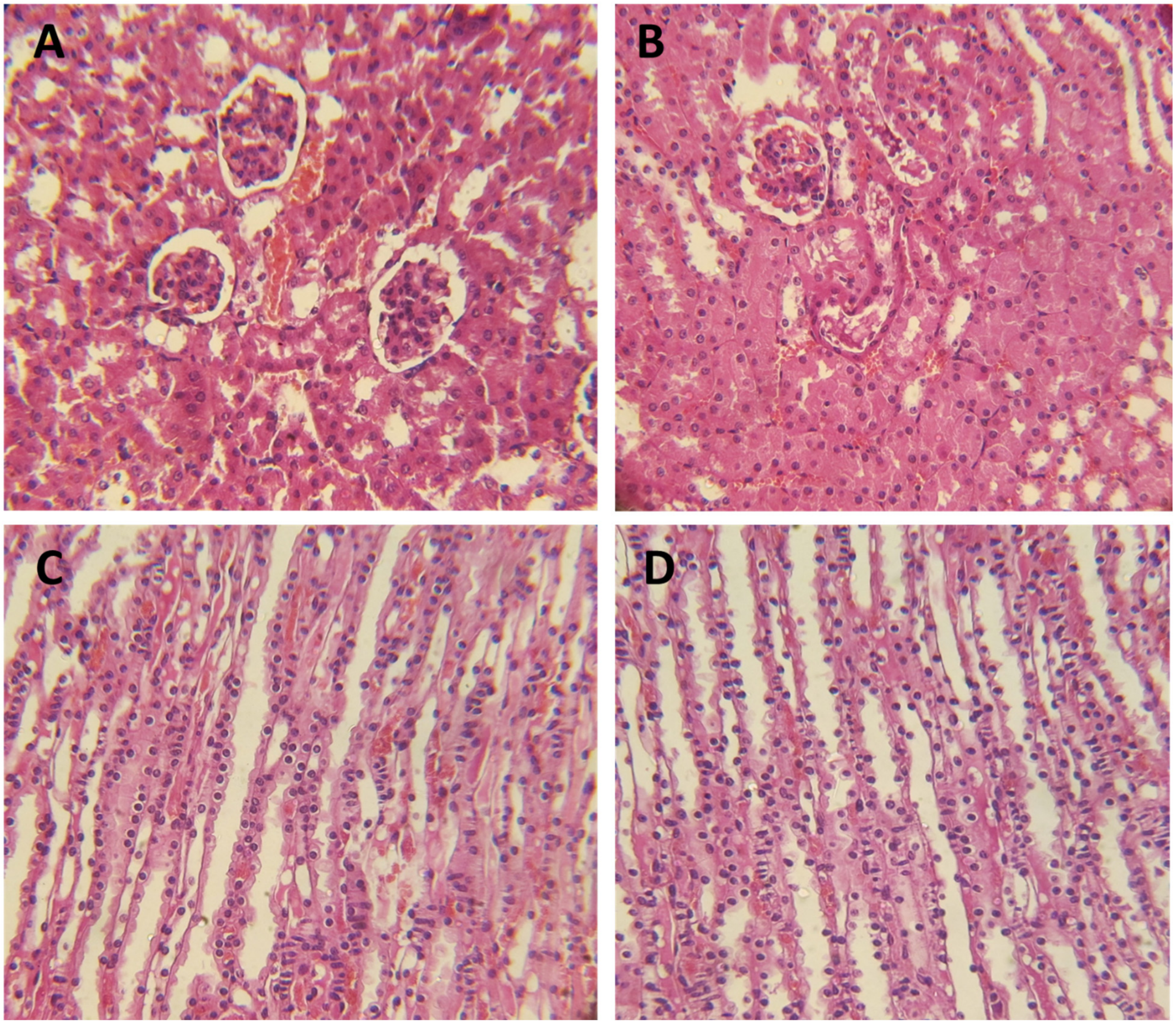

Figure S4. The histopathology evaluation of kidney. Pathological events including glomerular congestion, cloudy swelling of the tubules and glomerular necrosis, acute tubular necrosis, hemorrhage, and tubular distention were evaluated. Analysis of the results showed that administration of analgesic peptide induces no nephrotoxicity at the doses of 0.32 (**A**) and (**B**) and 0.64 mg/kg (**C**) and (**D**).

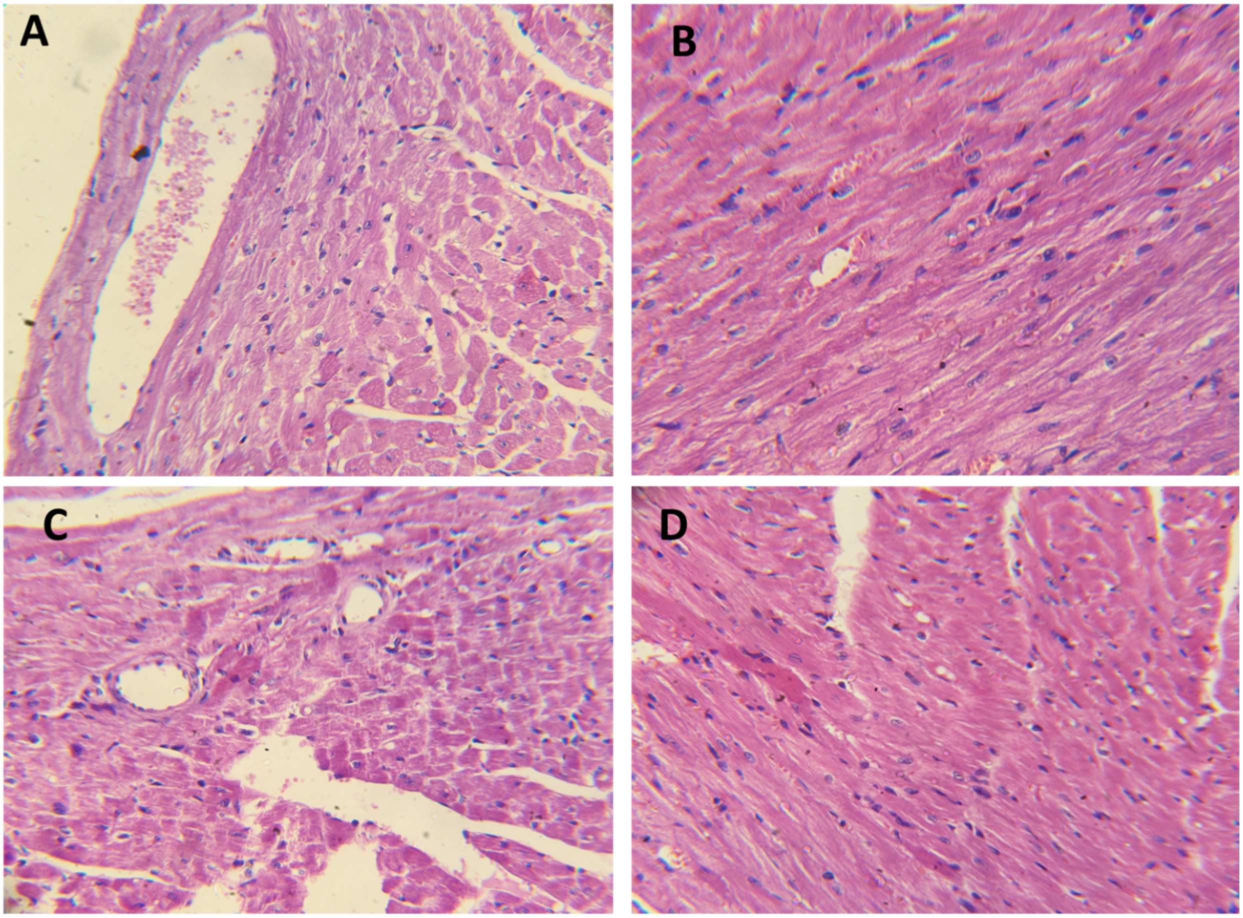

Figure S5. The histopathology evaluation of heart. Pathological events including Necrosis, hemorrhage, degeneration of nucleolus, piknotic nucleolus, edema, and granulation of nucleolus were evaluated. Analysis of the results showed that administration of analgesic peptide induces no cardiotoxicity at the doses of 0.32 (**A**) and (**B**) and 0.64 mg/kg (**C**) and (**D**).

Table S1. In vitro hemolysis assay for Leptucin.

	**Leptucin 8**	**Leptucin 16**	**Positive Control**	**Negative Control**
Hemolysis (%)	0	0	100	0

Table S2. In vivo hemolysis for Leptucin.

Test / Time	Leptucin (0.32 mg/kg)	Leptucin (0.48mg/kg)	Leptucin (0.64mg/kg)	Negative Control
24 h	0.2 ± 0.021	0.2 ± 0.025	0.2 ± 0.023	0.2 ± 0.023
48 h	0.2 ± 0.024	0.2 ± 0.015	0.2 ± 0.025	0.2 ± 0.026

Table S3. Determination of LD50 for Leptucin.

Dose (mg/kg)	Survived	Died	Total
0.8	6	0	6
1.6	6	0	6
2.4	6	0	6
3.2	6	0	6
4	6	0	6
All Doses	30	0	30
Estimated D50	>4 mg/kg		
